# 

**Published:** 1882-07

**Authors:** 


Vol. III.] TWO DOLLARS A YEAR. SINGLE COPIES 60 CENTS. [No. 3.

### THE JOURNAL

OF

### COMPARATIVE MEDICINE AND SURGERY.

S.G.O. INDEXED

### A Quarterly Journal

OF THE

### ANATOMY, PATHOLOGY, AND THERAPEUTICS OF THE LOWER ANIMALS.

NEW YORK:

W. L. HYDE & CO., PUBLISHERS, NO. 22 UNION SQUARE.

1882.

